# Breakfast Choice Is Associated with Nutrient, Food Group and Discretionary Intakes in Australian Adults at Both Breakfast and the Rest of the Day

**DOI:** 10.3390/nu11010175

**Published:** 2019-01-15

**Authors:** Flavia Fayet-Moore, Andrew McConnell, Tim Cassettari, Peter Petocz

**Affiliations:** 1 Nutrition Research Australia, Level 13 167 Macquarie Street, Sydney 2000, Australia; andrew@nraus.com (A.M.); tim@timcassettari.com (T.C.); 2 Department of Statistics, Macquarie University, Sydney 2109, Australia; peter.petocz@maquarie.edu.au

**Keywords:** breakfast, cereal, adult, diet, total day, BMI, Australia

## Abstract

Breakfast choice is correlated with daily nutrient intakes, but this association may not be solely explained by the breakfast meal. We profiled breakfast consumer groups among Australian adults and compared the role that breakfast versus the rest of the day had on daily intakes of the Five Food Groups, discretionary foods, and nutrients. Breakfast groups were breakfast cereal consumers, non-cereal breakfast consumers, and breakfast skippers. One-day dietary recall data from the 2011–2012 National Nutrition and Physical Activity Survey were analysed (*n* = 9341, ≥19 years), as well as socio-demographic and anthropometric measures. Twelve per cent of adults were breakfast skippers, 41% were breakfast cereal consumers, and 47% were non-cereal breakfast consumers. Females were more likely to have a non-cereal breakfast than males, and the non-cereal breakfast was predominantly bread-based. Breakfast skipping decreased with age (*p* < 0.001), while breakfast cereal consumption increased with age (*p* < 0.001). Breakfast skippers were more likely to be male, had a lower socio-economic status, and lower physical activity levels (*p* < 0.001). Breakfast skippers had the highest mean body mass index (BMI) and waist circumference (*p* < 0.001), the lowest intake of wholegrain foods, fruits and vegetables, and the highest intake of discretionary foods (*p* < 0.001). Breakfast cereal consumers had the lowest mean BMI and waist circumference (*p* < 0.001) and had healthier diets at both breakfast and throughout the rest of the day. They were the most likely to meet the daily recommended serves for grain foods, fruit, dairy, and vegetables, had the highest wholegrain food intake, and the lowest discretionary intake (*p* < 0.001). Additionally, breakfast cereal consumers had the most favourable daily nutrient intakes, including the lowest added sugars intakes. Differences in daily diet between breakfast groups were attributed to differences in food choices both at breakfast and throughout the rest of the day.

## 1. Introduction

Breakfast has been regarded as one of the most important meals of the day and breakfast consumption is associated with higher daily intake of dietary fibre and certain micronutrients [1,2], lower obesity prevalence [2], and fewer unhealthy lifestyle behaviours including smoking, alcohol use, and sedentary lifestyle [3]. Infrequent breakfast consumption and breakfast skipping have been associated with a higher prevalence of cardiovascular disease risk factors including hypertension and metabolic syndrome [4], and a greater risk of diabetes mellitus [5]. Clinical intervention studies have shown that a higher caloric intake in the morning may positively influence glucose and insulin metabolism [4]. In Australia, the most recent Australian Health Survey showed at least two in three adults did not meet the recommended number of serves for each of the Five Food Groups outlined in the Australian Dietary Guidelines (ADG) including fruit; vegetables and legumes/beans; and grain (cereal) foods [6]. At the same time, discretionary foods (those that provide little to no nutritional benefit to the diet) contributed more than one-third of total energy intake [7], many Australians exceeded the recommended targets for free sugars [8] and for sodium [9], and the proportion of Australian adults classified as overweight or obese has risen from 57% in 1995 to 63% in 2014–2015 [10]. 

Given the diet of Australian adults, and the rise in overweight and obesity, it is important to determine the relationship that breakfast choice has on dietary intake and anthropometric measures. Breakfast cereal consumption appears to be the key driver in the positive association between breakfast consumption and total daily nutrient intakes [11]. Studies in adults show that among breakfast consumers, those who consume cereal have greater intakes of fibre and micronutrients than those who consume other breakfasts (non-cereal breakfast consumers) [12,13,14]. This association is in part due to the fortification of many breakfast cereals, and also the inclusion of foods often consumed with breakfast cereal, such as milk [15]. Breakfast cereal consumption has also been associated with a lower or equivalent body mass index (BMI) or prevalence of obesity compared to other breakfast consumers [16,17] and non-consumers of breakfast [18,19]. Among children in Australia, those who consumed breakfast cereal had a higher intake of dietary fibre and micronutrients than those who had a non-cereal breakfast, with the exception of sodium, which was lower among breakfast cereal consumers [20,21,22].

It is possible that the favourable nutrient intakes of breakfast cereal consumers are confounded by dietary choices outside of the breakfast meal, but there are currently limited data to assess this. In a 2014 systematic review on breakfast cereals that included 21 cross-sectional studies on daily nutrient intakes in adults [11], only two studies assessed daily intakes throughout the day minus the breakfast meal [23,24]. Both of these studies were based on data that are at least 20 years old and neither study was Australian. Other potential confounders include the higher socio-economic status (SES) of breakfast cereal consumers [11]. The majority of research on the dietary associations of breakfast cereal consumption has analysed nutrient intakes [11,25], rather than the intake of foods or food groups. Yet dietary guidelines, including the ADG, are increasingly food-based [26,27,28]. 

The most recent breakfast consumption analysis to use nationally representative data among Australian adults is more than 20 years old [29]. Given the lack of recent analyses on breakfast choice, and the limited number of studies that examine breakfast intake compared to that of the rest of the day, there is a need to profile the diet of breakfast consumers and breakfast skippers, taking into account breakfast and the rest of the day’s intake, and capturing both nutrient and food group intake. We hypothesised that the breakfast cereal consumers would have a more favourable nutrient profile at breakfast compared to non-cereal breakfast consumers, driven by food choices made at breakfast, and that daily differences may not be explained by breakfast choices alone.

The aim of this study was therefore to profile the socio-demographic, anthropometric and dietary characteristics of breakfast consumer groups (breakfast skippers, cereal breakfast, and non-cereal breakfast), and to compare the impact of breakfast versus the food consumed during the rest of the day on their diets.

## 2. Materials and Methods

### 2.1. Survey Methodology

Data from the 2011–2012 National Nutrition and Physical Activity Survey (NNPAS) were used. The NNPAS was a nationally representative survey performed by the Australian Bureau of Statistics (ABS) as part of the 2011–2013 Australian Health Survey. Households from all Australian states and territories were selected using a stratified multi-stage area sampling plan. Dwellings from Very Remote areas and discrete Aboriginal and Torres Strait Islander communities were excluded, as well as non-private dwellings such as caravan parks. There were no exclusion criteria based on health indicators. One adult and one child per household were randomly selected for the survey. Trained ABS interviewers collected data from 12,153 participants during face-to-face interviews between May 2011 and June 2012. An Automated Multiple-Pass Method [30] was used to capture foods and beverages consumed by respondents during the 24 h prior to the interview day. Interviewers used set questions to acquire details on food types and a Food Model Booklet [31] to determine portion sizes. Approximately two-thirds of respondents provided a second day of dietary recall via telephone interview. For this study, to maximise the sample size, only day one of dietary recall was examined from the 9341 respondents aged 19 years and over. Weightings provided by the ABS were used to weight the data to represent the Australian population. Interview components of the survey were conducted under the Census and Statistics Act 1905. Ethics approval was not necessary.

### 2.2. Breakfast and Breakfast Cereal Intake

As part of the 24-h dietary recall, respondents stated the eating occasion for foods and beverages consumed from 11 options: breakfast, brunch, morning tea, lunch, afternoon tea, dinner, supper, snack, beverage/drink, extended consumption, and other. We defined breakfast as any food or beverage consumed at the “breakfast” eating occasion, regardless of time of day. Any food from the sub-major food groups “Breakfast cereals, ready to eat” or “Breakfast cereals, hot porridge style” was also classified as breakfast if it was reported as an “extended consumption” eating occasion, and was consumed between 5.30 a.m. and 9.30 a.m. Foods not defined as breakfast were classified as being consumed during “the rest of the day”.

Each respondent was classified as a “breakfast skipper” or “breakfast consumer”, and breakfast consumers were classified as a “non-cereal breakfast consumer” or a “breakfast cereal consumer”. Breakfast cereal consumers were also classified based on the total sugars content of the cereal. A minimally pre-sweetened (MPS) consumer was an exclusive consumer of MPS cereal that contained <15% total sugars (<15 g/100 g). Consumers of pre-sweetened (PS) cereal were classified according to the level of pre-sweetening: a PS15 consumer had cereal that was ≥15% and <30% total sugars (including those who also consumed MPS cereal), and a PS30 consumer had cereal that was ≥30% total sugars (including those who also consumed MPS and/or PS15 cereal).

“Breakfast cereal” was defined as any food from the sub-major food groups “Breakfast cereals, ready to eat” or “Breakfast cereals, hot porridge style” consumed as breakfast. A non-cereal breakfast consumer was a breakfast consumer who did not have breakfast cereal, and a breakfast skipper was a respondent who did not have breakfast.

### 2.3. Dietary and Nutrient Intakes

Dietary intake data were calculated from the survey-specific 2011–2013 Australian Food Composition Database (AUSNUT) developed by Food Standards Australia New Zealand (FSANZ), which contains nutrient contents of over 5000 foods and beverages [32]. Foods and beverages were categorised into 132 sub-major food groups that specify the key ingredient and characteristics of that ingredient, such as plant types or cooking variations. The top 10 breakfast sub-major food groups were determined for breakfast cereal consumers and non-cereal breakfast consumers, ranked by the prevalence of consumers, and the mean and median intake in grams of each sub-major food group at breakfast was calculated. Daily energy and nutrient intakes were calculated for all respondents, as well as intakes at breakfast and during the rest of the day. The total intakes and proportion of daily energy and nutrients from breakfast cereal were calculated. The macronutrients investigated were protein; total and saturated fat; total, added, and free sugars; carbohydrates; and fibre. The micronutrients were niacin, iron, thiamin, riboflavin, folate, calcium, sodium, magnesium, and potassium.

### 2.4. Discretionary Foods

The ADG defines discretionary foods as foods and drinks not necessary for a nutritious diet, high in saturated fat, added sugars, added salt, or alcohol [33]. Foods and drinks in the NNPAS were classified as core (non-discretionary) or discretionary by the ABS. Breakfast cereal was defined as a discretionary food if it contained >30 g/100 g total sugars or >35 g/100 g total sugars for cereal with added fruit. The total serves of discretionary foods (1 serve = 600 kJ) were calculated throughout the day, at breakfast, and during the rest of the day. The top 10 discretionary sub-major food groups consumed at breakfast were determined by breakfast consumer group. The per cent energy contribution of these food groups at breakfast to total daily discretionary energy was calculated, as well as the per cent of each breakfast consumer group that consumed the food group, and the mean kJ among those consumers.

### 2.5. Australian Dietary Guidelines Five Food Groups

The ADG includes the Australian Guide to Healthy Eating, which advises the consumption of the Five Food Groups [28]: grain (cereal) foods, mostly wholegrain and/or high cereal fibre varieties, such as bread, cereals, rice, pasta, noodles, polenta, couscous, oats, quinoa, and barley (“grain foods”); plenty of vegetables, including different types and colours, and legumes/beans (“vegetables”); fruit; milk, yoghurt, cheese and/or their alternatives, mostly reduced fat (“dairy”); and lean meats and poultry, fish, eggs, tofu, nuts and seeds and legumes/beans (“meat”). Grain foods were further defined as either wholegrain or higher fibre cereals/grains (“wholegrain foods”) or refined or lower fibre cereals/grains (“refined grain foods”). Discretionary foods are excluded from the Five Food Groups. The total serves at breakfast, during the rest of the day, and throughout the entire day for each of the Five Food Groups were calculated using a database created by the ABS and FSANZ which lists the amount of the food groups contained in the AUSNUT foods and beverages [34]. The proportion of participants that met the recommended serves of each of the Five Food Groups by breakfast consumer category was calculated.

### 2.6. Demographic and Anthropometric Measures

Socio-economic status (SES) is based on the Socio-Economic Indexes for Areas (SEIFA) [35]. SEIFA is an ABS product that ranks Australian areas into quintiles according to relative socio-economic advantage or disadvantage. The lowest quintile was defined as the first quintile, and the highest as the fifth quintile.

Respondents’ weight, height, and waist circumference were measured during the face-to-face interview. The body mass index (BMI, kg/m^2^) was calculated using participants’ height and weight and they were classified as: underweight (<18.5 kg/m^2^), normal weight (≥18.5 kg/m^2^ to <25 kg/m^2^), overweight (≥25 kg/m^2^ to <30 kg/m^2^), or obese (≥30 kg/m^2^) [36]. Participants’ waist circumference defined their level of risk of metabolic complications based on the World Health Organisation categories: not at risk (<80 cm females, <94 cm males), at increased risk (≥80 cm to <88 cm females, ≥94 cm to <104 cm males), or at substantially increased risk (≥88 cm females, ≥104 cm males) [37].

### 2.7. Statistical Analyses

The statistical platform IBM SPSS version 25.0 (IBM Corp., Armonk, NY, USA) was used for all analyses, and due to the large sample size (*n* = 9341), *p*-values < 0.001 were treated as significant. Pearson chi-square tests were used to examine associations between categories of breakfast consumers and demographic, anthropometric, and physical activity measures. Associations between the prevalence of adults meeting the recommended serves of the Five Food Groups and breakfast consumer category were also tested using Pearson chi-square tests. 

Marginal means for BMI and waist circumference were calculated using general linear models adjusted for confounding factors. Intake of the Five Food Groups and nutrients by breakfast consumer category were also investigated using general linear models. Post-hoc comparisons with Bonferroni adjustment were used to investigate pairwise significance between categories of breakfast consumers for all general linear models performed.

## 3. Results

### 3.1. Demographic, Anthropometric, and Lifestyle Characteristics

One in eight adults (12%) skipped breakfast, and there were more male skippers (14%) than female (11%) (Table 1). Among those who consumed breakfast, almost half had breakfast cereal (46%), and the majority of them consumed ready-to-eat cereal (82%) and dairy milk (79%) at breakfast (Supplementary Table S1). Almost two-thirds (62%) of breakfast cereal consumers were MPS consumers, 35% were PS15 consumers, and 3% were PS30 consumers. The non-cereal breakfast was predominantly bread-based (52%), and contained dairy milk but at a lower prevalence (36%) and spreads (including butter, margarine and yeast extract). Females were more likely to have a non-cereal breakfast than males (49% vs. 45%, respectively) (*p* < 0.001) and breakfast skipping decreased with age, while breakfast cereal consumption increased with age (*p* < 0.001). 

Breakfast cereal consumers had the lowest mean BMI, waist circumference, and prevalence of overweight and obesity across breakfast consumption categories (*p* < 0.001), but there was no difference in the prevalence of being at risk of metabolic complications based on waist circumference. They were the most likely to be sufficiently active for health and had the highest prevalence of being from the highest SES quintile (*p* < 0.001).

### 3.2. Recommended Serves of ADG Five Food Groups

Approximately one-third of adults met the ADG recommended serves of grain foods, fruit, and meat, and one in seven adults met the recommended serves of vegetables and dairy (Table 2). Breakfast cereal consumers were the most likely to meet the recommended serves of grain foods (41%), fruit (36%), vegetables (18%), and dairy (20%) (*p* < 0.001). Breakfast skippers were the least likely to consume sufficient serves of grain foods (18%), fruit (18%), and vegetables (9%), while non-cereal breakfast consumers had the lowest prevalence of meeting the recommended serves of dairy (11%) (*p* < 0.001). There was no difference in the prevalence of meeting the targets for meat between breakfast consumer categories.

### 3.3. Intakes of ADG Five Food Groups per Day, at Breakfast, and During the Rest of the Day

Breakfast cereal consumers had the highest daily serves of grain foods, wholegrain foods, fruit, and dairy and the lowest intake of refined grain foods (*p* < 0.001) (Table 3). Daily wholegrain food serves among breakfast cereal consumers were double that of non-cereal breakfast consumers and triple that of breakfast skippers (*p* < 0.001). Breakfast cereal consumers had similar daily intakes of wholegrain foods and refined grain foods, whereas non-cereal breakfast consumers had twice as much refined grain foods as they had wholegrain foods, and breakfast skippers had more than three times as much refined grain foods as wholegrain foods. The greater wholegrain food intake among breakfast cereal consumers was mostly driven by breakfast: they had 1.1 more serves of wholegrain foods at breakfast compared to non-cereal breakfast consumers, versus 0.2 more wholegrain food serves throughout the rest of the day (*p* < 0.001). Fruit and dairy serves at both breakfast and throughout the rest of the day were highest for breakfast cereal consumers (*p* < 0.001) and breakfast cereal consumers had double the fruit and dairy intake at breakfast compared to non-cereal breakfast consumers (*p* < 0.001). Breakfast skippers had the lowest daily intake of grain foods, wholegrain foods, fruit and vegetables (*p* < 0.001).

There was no difference in daily vegetable serves between breakfast cereal consumers and non-cereal breakfast consumers, despite a higher intake of vegetable serves among non-cereal breakfast consumers at breakfast, but a lower intake during the rest of the day (*p* < 0.001). Daily meat serves were higher among non-cereal breakfast consumers compared to breakfast cereal consumers, which was driven by their intake at breakfast (*p* < 0.001).

### 3.4. Intakes of Discretionary Foods

Breakfast skippers had the highest daily serves of discretionary foods (6.3 serves) and breakfast cereal consumers had the lowest (4.5 serves) (*p* < 0.001) (Table 3). Non-cereal breakfast consumers had more than three times the discretionary intake at breakfast than breakfast cereal consumers (*p* < 0.001), and a higher intake during the rest of the day (*p* < 0.001). The top discretionary sub-major food group for both breakfast cereal consumers and non-cereal breakfast consumers at breakfast was “sugar, honey and syrups” (Supplementary Table S2). In breakfast cereal consumers, “sugar, honey and syrups” was the major contributor to daily discretionary energy, as it contributed more than the next nine food groups combined.

### 3.5. Daily Energy and Nutrient Intakes

Breakfast skippers had the lowest daily energy intake (*p* < 0.001) (Table 4). Breakfast cereal consumers had the lowest intake of total and saturated fat, and added and free sugars (*p* < 0.001). Breakfast cereal consumers had the highest intake of carbohydrate and fibre, a higher intake of protein compared to breakfast skippers, and a higher intake of total sugars compared to non-cereal breakfast consumers (*p* < 0.001). Breakfast skippers had the highest intake of added and free sugars of all groups (*p* < 0.001).

Breakfast cereal consumers had the highest daily intakes of all micronutrients analysed apart from sodium, for which non-cereal breakfast consumers had the highest intake (*p* < 0.001). Breakfast skippers had a similar daily intake of niacin to non-cereal breakfast consumers and a similar intake of sodium to breakfast cereal consumers, and the lowest daily intake of all other micronutrients analysed (*p* < 0.001). Non-cereal breakfast consumers had the highest daily sodium intake of all groups (*p* < 0.001).

There was no difference in intakes of added and free sugars at breakfast between breakfast cereal and non-cereal consumers, but breakfast cereal consumers had the lowest intakes of all groups during the rest of the day (*p* < 0.001). Higher daily fibre intake among breakfast cereal consumers was driven by a greater intake at breakfast and during the rest of the day, compared to non-cereal breakfast consumers (*p* < 0.001).

Compared to non-cereal breakfast consumers, breakfast cereal consumers had a higher intake at breakfast of energy and all micronutrients analysed apart from sodium, which was lower. During the rest of the day, breakfast cereal consumers had higher intakes of iron, riboflavin, folate, calcium, magnesium, and potassium compared to non-cereal breakfast consumers (*p* < 0.001), but there was no difference in intakes of niacin, thiamin, and sodium.

Relative to its energy contribution, breakfast cereal contributed half the total fat, almost one and a half times as much carbohydrate, half the sodium, at least double the fibre, riboflavin, and folate, and more than triple the iron and thiamin (Supplementary Table S3).

## 4. Discussion

In this nationally representative sample of Australian adults, the majority (88%) had breakfast, and almost half of those had a breakfast cereal breakfast. Breakfast skippers were younger, more likely to be male, had a higher mean BMI and waist circumference, lower physical activity level and lower SES. Breakfast cereal consumers were older, had the lowest mean BMI and waist circumference, and were more likely to be physically active and socio-economically advantaged. Breakfast cereal consumers had a healthier diet at both breakfast and throughout the rest of the day, being the most likely to meet the daily recommended serves for grain foods, fruit, dairy, and vegetables and having the lowest discretionary intake; whereas breakfast skippers had the lowest intake of wholegrain foods, fruits and vegetables, and highest discretionary intake. Consequently, breakfast cereal consumers had the most favourable nutrient intakes and breakfast skippers the least favourable. 

Breakfast and breakfast cereal consumption in our study were popular in comparison to other nutrition surveys globally, although direct comparisons are limited due to differences in how breakfast is defined, survey methodologies, and the adult age groups included. In Canada, the prevalence of breakfast skipping was similar (11%), but a non-cereal breakfast was more popular, with 69% of adults being “other” breakfast consumers and 20% ready-to-eat breakfast cereal consumers [12]. Similarly, in the U.S., 17% consumed ready-to-eat cereal, 63% other breakfasts, but breakfast skipping prevalence was higher (20%) [38]. In a study of 19–64-year-old adults in Britain, breakfast skipping was also more prevalent (22%) than in Australia, but 33% consumed breakfast cereal for breakfast, and 45% a non-cereal breakfast [14]. The most recently published data among adults in Australia was from the 1995 National Nutrition Survey, and it reported that 77% of adults consumed breakfast five or more times per week [39]. 

There were clear associations between breakfast consumer groups with sex, age, SES, physical activity, BMI, and waist circumference. Our findings are consistent with a large body of observational evidence showing that breakfast skippers are younger [39,40] and have less healthy lifestyles that include poorer quality diets and lower physical activity levels [1,3,41,42,43,44]. Targeted breakfast interventions may be particularly important for young adult males who also have poorer diets, including lower vegetable intake [45], higher discretionary intake [46], and lower diet quality [47]. In line with our findings, previous research consistently reports that adult breakfast cereal consumers tend to be older [12,48,15] and have a lower BMI [16,17,18,19] compared to those who do not consume breakfast cereal. Since the data are observational, confounding or reverse causality could explain the associations between breakfast group and anthropometric measures. We also found that breakfast cereal consumers had the highest SES. A large body of evidence shows that SES is correlated with breakfast consumption [49], diet quality [50,51], including in Australia [47], and it may be that breakfast cereal consumption may be a marker of greater availability, accessibility or spending capacity for nutrient-rich food. Intervention studies on breakfast and breakfast cereal consumption, across a range of different socio-demographic groups, would determine the nature of these associations.

Our findings on diet are in line with previous comparative analyses of breakfast cereal consumers, non-cereal breakfast consumers and breakfast skippers, that showed breakfast cereal consumers tend to have the most favourable daily nutrient intakes, and breakfast skippers the least favourable, both in children [14,20,21,22,52,53,54,55,56,57,58,59,60] and in adults [12,13,24]. These data are more limited for food groups, but those that have analysed food group intakes showed that breakfast cereal consumers had higher intakes of grain foods, dairy or fruit compared to other breakfast consumers [13,52,61,62], consistent with our findings. These results are important since the majority of Australian adults did not meet the daily targets for each of the Five Food Groups.

In our study, the intake of wholegrain foods, dairy and fruit were higher among breakfast cereal consumers compared to non-cereal breakfast consumers both at breakfast, and throughout the rest of the day, with differences larger at breakfast. Thus, differences throughout the day were explained to a greater extent by the breakfast meal. The higher wholegrain food intake at breakfast is likely due to the contribution of wholegrain foods from the breakfast cereal. About two in three (70%) Australian breakfast cereals contained ≥8 g of wholegrain per serve, 85% of breakfast cereals contained at least 2 g of fibre per serve [63], and breakfast cereal was the leading contributor to wholegrain food intake in the Australian diet [64]. In the U.S., modelling evidence showed that the replacement of a non-cereal breakfast with breakfast cereal increased wholegrain intake among children and adults [65]. Refined grain food intake at breakfast was higher in non-cereal breakfast consumers which was likely due to bread being consumed by more than half of these consumers, with white bread the leading type of bread consumed in the Australian diet [7].

With regards to dairy, the prevalence of dairy milk consumption among breakfast cereal consumers in our study was more than double that of non-cereal breakfast consumers, consistent with previous cross-sectional research in the U.S. that showed ready-to-eat cereal was associated with enhanced milk intake in all age and sex groups compared to other breakfasts [15]. We also found that breakfast cereal consumers had five times more milk per consumer than non-cereal breakfast consumers, which may be due to how it was consumed (e.g., with breakfast cereal vs. in tea or coffee). Thus, the higher dairy intake among breakfast cereal consumers at breakfast was a result of both the higher number of milk consumers and the greater intake of milk per consumer. Our findings suggest that non-cereal breakfast choices may be missed opportunities to consume dairy, as well as wholegrain foods and fruit, which could negatively impact total daily intakes.

Breakfast cereal consumers had a lower intake of vegetables and meat at breakfast, consistent with a previous analysis of young adults in the U.S. that showed ready-to-eat cereal breakfast consumers had a lower intake of total vegetables and meat/poultry/fish at breakfast compared with other breakfast consumers [13]. Despite not having vegetables at breakfast, the vegetable intake of breakfast cereal consumers was higher throughout the rest of the day, which is evidence of a generally healthier dietary intake on that day. Thus, breakfast recommendations may need to be made in the context of the total diet.

Across all breakfast consumer groups, fibre and micronutrient intakes followed the same pattern as their intake of the ADG Five Food Groups. This is expected since the food group recommendations in the ADG are set to ensure nutrient needs are met. The significance of breakfast choice on meeting targets is highlighted by the higher breakfast intakes of under-consumed nutrients in breakfast cereal consumers compared to non-cereal breakfast consumers, including fibre, calcium and iron. The higher fibre intake among breakfast cereal consumers may be driven by the higher grain food (including wholegrain food) and fruit intake at breakfast since vegetable intake was lower. Differences in calcium are likely due to the higher milk and dairy intake among breakfast cereal consumers, with dairy milk the leading contributor of calcium among Australian adults [7]. Of interest, iron intake was highest in breakfast cereal consumers despite meat intake being lower in this group. With breakfast cereal contributing a third of daily iron among breakfast cereal consumers, the higher iron intake may be due to the fortification of breakfast cereals, since ready-to-eat breakfast cereals was the leading contributor to iron in the diet of Australian adults [7], and the consumption of a fortified cereal has been shown to increase both iron intake and status [66].

Breakfast skippers had the highest daily discretionary intake, in line with previous findings on breakfast skipping in Australian children [67]. Among those who had breakfast, non-cereal consumers had a higher discretionary intake at breakfast compared to cereal consumers, who also had a lower discretionary intake throughout the rest of the day. These findings are important given discretionary foods contribute more than a third of energy intake in the Australian diet [7] and more than 80% of added and free sugars [68]. Breakfast cereals contributed <3% to the total free and added sugars intake in adults [8], which may be partly due to the fact that nearly two in three adults had a cereal with lower total sugars in our study. We found no differences in added or free sugars intakes at breakfast among the two breakfast groups, though breakfast cereal consumers had lower intakes of added and free sugars throughout the rest of the day and consequently the lowest daily added and free sugars intake. This is in contrast to evidence in U.S. adults [13], where no differences in the total daily intakes were reported. Breakfast cereal consumers had less than half the sodium intake at breakfast compared to non-cereal breakfast consumers, who had the highest daily sodium intakes despite no differences throughout the rest of the day. This suggests that breakfast may be driving the higher sodium intake of non-cereal breakfast consumers, which is not surprising given that some of the popular non-cereal breakfast choices are higher in sodium, including bread, yeast-based spreads and processed meats.

The association with lower discretionary intake and lower sugars and sodium intakes throughout the rest of the day may be in part due to breakfast cereal consumers being more likely to make healthier dietary choices throughout the rest of the day (and breakfast skippers less healthy dietary choices), or it may be due to the causal influence that breakfast choice may have on subsequent consumption during the rest of the day. Importantly, both non-cereal breakfasts [38] and breakfast cereal breakfast [69] are not homogenous, and this may influence the relationship between breakfast choice and dietary intake. Breakfast cereals range in their nutrient profile including added sugars, sodium and fortified nutrients like iron and calcium [70]. The ADG recommends that consumers choose those breakfast cereals that are sources of wholegrain or high in cereal fibre and lower in added sugars and added salt. Accordingly, there is evidence of alignment of breakfast cereals with the AGD, where manufacturers have reformulated some of their products to meet sodium reduction targets [71,72].

Strengths of our study include the use of a large, nationally representative sample of the Australian adult population. In addition to daily intakes, our analysis separated intakes at the breakfast meal and throughout the rest of the day to provide an insight into how these different eating occasions and intakes were independently associated with breakfast consumer group. Unlike other Australian studies on breakfast, in addition to nutrient intakes we also analysed associations with the ADG Five Food Groups. Our study has limitations. Dietary intake was derived from a single day of 24-h recall and is not necessarily indicative of usual intakes. Two-thirds of respondents in the NNPAS provided two days of recall, however, one day of recall was used in order to maximise the sample size. Under-reporting was common in the NNPAS [32] and it is possible that breakfast skippers, who had the lowest daily energy intake, were more likely to underreport. This group, however, had the highest discretionary intake, and it has been shown that discretionary foods are more likely to be underreported than other foods [73]. Finally, the data are cross-sectional, precluding causal relationships.

## 5. Conclusions

Breakfast cereal consumers had a healthier diet including a greater likelihood of meeting the ADG Five Food Group recommendations, lower intakes of refined grain foods, discretionary foods, added sugars and sodium, and higher intakes of wholegrain foods, fibre, and micronutrients. Breakfast cereal consumers also had a lower BMI and waist circumference. In contrast, breakfast skippers had the most unfavourable dietary associations. Dietary differences between breakfast consumers groups were explained not only by the breakfast meal but also by food choices throughout the rest of the day. Differences in anthropometric measures may be due to the healthier diet but also confounding factors, with other differences in lifestyle and socioeconomic factors found. In conclusion, a breakfast cereal breakfast may help to improve the likelihood of meeting ADG Five Food Groups recommendations and increase intake of under-consumed nutrients, without increasing discretionary energy intake. Further research including intervention studies is required to determine causality.

## Figures and Tables

**Table 1:** Socio-demographic, anthropometric, and physical activity measures by breakfast consumer category.

**Characteristic**	**Breakfast Cereal Consumers ^1^**	**Non-Cereal Breakfast Consumers ^2^**	**Breakfast Skippers ^3^**	*p* Value from Pearson’s Chi-Square Test *
N (%)	3798 (40.7%)	4392 (47.0%)	1151 (12.3%)	
Age (years ± SE)	50.2 ± 0.3 ^a^	44.9 ± 0.2 ^b^	38.7 ± 0.4 ^c^	<0.001
Age group (% within age group)				<0.001
19–30 years	32.2%	46.6%	21.2%	
31–50 years	36.2%	51.5%	12.3%	
51–70 years	45.3%	46.0%	8.7%	
71+ years	61.9%	35.1%	3.1%	
Sex (% within sex group)				<0.001
Male	41.2%	44.7%	14.1%	
Female	40.1%	49.3%	10.6%	
BMI group ^†^ (% within breakfast category)				<0.001
Underweight	1.2%	2.2%	2.3%	
Normal	37.2%	34.7%	32.7%	
Overweight	36.6%	36.5%	35.6%	
Obese	25.0%	26.6%	29.5%	
Waist group ^‡^ (% within breakfast category)				0.268
Not at risk of metabolic complications	36.9%	37.5%	38.8%	
Increased risk of metabolic complications	24.0%	22.4%	20.8%	
Substantially increased risk of metabolic complications	39.1%	40.1%	40.4%	
SES quintile ^§^ (% within breakfast category)				<0.001
Lowest 20%	16.1%	18.7%	22.0%	
Second quintile	18.6%	20.4%	23.8%	
Third quintile	20.9%	20.9%	18.6%	
Fourth quintile	19.9%	18.5%	17.9%	
Highest 20%	24.4%	21.5%	17.7%	
Whether participated in sufficient activity in last week ^||^ (% within breakfast category)				<0.001
Inactive	18.6%	20.6%	27.7%	
Insufficiently active	35.7%	36.4%	35.3%	
Sufficiently active for health	45.7%	43.0%	37.0%	
**Characteristic**	**Breakfast Cereal Consumers ^1^**	**Non-Cereal Breakfast Consumers ^2^**	**Breakfast Skippers ^3^**	*p* Value **
BMI ^¶^ (kg/m^2^ ± SE)	26.9 ± 0.1 ^a^	27.4 ± 0.1 ^b^	28.0 ± 0.2 ^b^	<0.001
Waist circumference ^¶^ (cm ± SE)	92.5 ± 0.2 ^a^	93.8 ± 0.2 ^b^	94.8 ± 0.4 ^b^	<0.001

Abbreviations: SE—standard error; BMI—body mass index; SES—socio-economic status. ^1^ Breakfast cereal consumers had any food from the sub-major food groups “breakfast cereals, ready to eat” or “breakfast cereals, hot porridge style”, either at any time of day with a “breakfast” eating occasion, or between 5.30 a.m. and 9.30 a.m. with an “extended consumption” eating occasion. ^2^ Non-cereal consumers reported “breakfast” eating occasions but were not breakfast cereal consumers. ^3^ Breakfast skippers did not report a “breakfast” eating occasion. ^a,b,c^ Different superscripts denotes significant difference between groups (*p* < 0.001) by post hoc, Bonferroni. * *p* values show associations between breakfast consumer categories and demographic, anthropometric, and lifestyle measures. ** Univariate ANOVAs denote the effect of breakfast consumer category on anthropometric measures. ^†^ Based on BMI: underweight (<18.5), normal weight (≥18.5, <25.0), overweight (≥25.0, <30.0), obese (≥30.0). ^‡^ Based on World Health Organization cut-offs for waist circumference: not at risk of metabolic complications (females: <80 cm; males: <94 cm); increased risk of metabolic complications (females: ≥80 cm, <88 cm; males: ≥94 cm, <102 cm); substantially increased risk of metabolic complications (females: >88 cm; males: >102 cm). ^§^ Based on Socio-Economic Indexes for Areas (SEIFA), a product developed by the ABS that ranks areas in Australia according to their relative socio-economic advantage. ^||^ For adults 18 years and over at least 150 min of physical activity over five or more sessions per week is recommended. ^¶^ Adjusted for age group, sex, physical activity, and energy intake.

**Table 2:** Prevalence of adults meeting the recommended serves of the Five Food Groups by breakfast consumer category.

Food Group *	All Adults (*n* = 9341)	Breakfast Cereal Consumers ^1^ (*n* = 3798)	Non-Cereal Breakfast Consumers ^2^ (*n* = 4392)	Breakfast Skippers ^3^ (*n* = 1151)	*p* Value
Grain foods ^4^	31.8%	41.0%	27.4%	17.9%	<0.001
Fruit ^5^	29.4%	35.9%	26.8%	17.7%	<0.001
Vegetables ^6^	15.7%	17.7%	15.7%	9.1%	<0.001
Meat ^7^	35.5%	35.0%	36.2%	34.3%	0.366
Dairy ^8^	14.7%	19.8%	10.9%	12.2%	<0.001

* Australian Dietary Guidelines Five Food Groups. ^1^ Breakfast cereal consumers had any food from the sub-major food groups “breakfast cereals, ready to eat” or “breakfast cereals, hot porridge style”, either at any time of day with a “breakfast” eating occasion, or between 5.30 a.m. and 9.30 a.m. with an “extended consumption” eating occasion. ^2^ Non-cereal consumers reported “breakfast” eating occasions but were not breakfast cereal consumers. ^3^ Breakfast skippers did not report a “breakfast” eating occasion. ^4^ Grain (cereal) foods, mostly wholegrain and/or high cereal fibre varieties. ^5^ Includes fruit or vegetable juice. ^6^ Vegetables and legumes/beans. ^7^ Lean meats and poultry, fish, eggs, tofu, nuts and seeds and legumes/beans. ^8^ Milk, yoghurt, cheese and/or alternatives, mostly reduced fat.

**Table 3:** Serves of the Five Food Groups and discretionary foods at breakfast, the rest of the day, and the total day by breakfast consumer category.

Food Group *	Total Day			Breakfast		The Rest of the Day		
	Breakfast Cereal Consumers ^1^ (*n* = 3798)	Non-Cereal Breakfast Consumers ^2^ (*n* = 4392)	Breakfast Skippers ^3^ (*n* = 1151)	Breakfast Cereal Consumers ^1^ (*n* = 3798)	Non-Cereal Breakfast Consumers ^2^ (*n* = 4392)	Breakfast Cereal Consumers ^1^ (*n* = 3798)	Non-Cereal Breakfast Consumers ^2^ (*n* = 4392)	Breakfast Skippers ^3^ (*n* = 1151)
	Serves ^†^ (mean ± SE)							
Grain foods ^4^	5.2 ± 0.1 ^a^	4.4 ± 0.1 ^b^	3.8 ± 0.1 ^c^	2.1 ± 0.0 ^a^	1.4 ± 0.0 ^b^	3.1 ± 0.1 ^a^	3.1 ± 0.1 ^a^	3.6 ± 0.1 ^b^
*Whole*	2.5 ± 0.0 ^a^	1.3 ± 0.0 ^b^	0.8 ± 0.1 ^c^	1.7 ± 0.0 ^a^	0.6 ± 0.0 ^b^	0.8 ± 0.0 ^a^	0.6 ± 0.0 ^b^	0.6 ± 0.0 ^b^
*Refined*	2.6 ± 0.1 ^a^	3.1 ± 0.1 ^b^	3.0 ± 0.1 ^c^	0.4 ± 0.0 ^a^	0.7 ± 0.0 ^b^	2.3 ± 0.1 ^a^	2.4 ± 0.1 ^a^	3.0 ± 0.1 ^b^
Fruit ^5^	1.8 ± 0.0 ^a^	1.4 ± 0.0 ^b^	1.1 ± 0.1 ^c^	0.6 ± 0.0 ^a^	0.3 ± 0.0 ^b^	1.3 ± 0.0 ^a^	1.1 ± 0.0 ^b^	1.1 ± 0.1 ^a, b^
Vegetables ^6^	3.2 ± 0.1 ^a^	3.1 ± 0.1 ^a^	2.7 ± 0.1 ^b^	0.0 ± 0.0 ^a^	0.2 ± 0.0 ^b^	3.2 ± 0.1 ^a^	2.8 ± 0.0 ^b^	2.7 ± 0.1 ^b^
Meat ^7^	2.0 ± 0.0 ^a^	2.2 ± 0.0 ^b^	2.3 ± 0.1 ^a,b^	0.1 ± 0.0 ^a^	0.2 ± 0.0 ^b^	2.0 ± 0.0 ^a^	2.0 ± 0.0 ^a^	2.3 ± 0.1 ^b^
Dairy ^8^	1.8 ± 0.0 ^a^	1.2 ± 0.0 ^b^	1.1 ± 0.0 ^b^	0.8 ± 0.0 ^a^	0.3 ± 0.0 ^b^	1.0 ± 0.0 ^a^	0.9 ± 0.0 ^b^	1.2 ± 0.0 ^a^
Discretionary foods	4.5 ± 0.1 ^a^	5.5 ± 0.1 ^b^	6.3 ± 0.1 ^c^	0.2 ± 0.0 ^a^	0.7 ± 0.0 ^b^	4.6 ± 0.1 ^a^	5.0 ± 0.1 ^b^	5.2 ± 0.1 ^b^

Abbreviations: SE—standard error. * Australian Dietary Guidelines Five Food Groups. ^†^ Adjusted for sex, age group, their interaction, BMI group, and energy intake. ^1^ Breakfast cereal consumers had any food from the sub-major food groups “breakfast cereals, ready to eat” or “breakfast cereals, hot porridge style”, either at any time of day with a “breakfast” eating occasion, or between 5.30 a.m. and 9.30 a.m. with an “extended consumption” eating occasion. ^2^ Non-cereal consumers reported “breakfast” eating occasions but were not breakfast cereal consumers. ^3^ Breakfast skippers did not report a “breakfast” eating occasion. ^a,b,c^ Different superscripts denotes significant difference between groups (*p* < 0.001) by post hoc, Bonferroni. ^4^ Grain (cereal) foods, mostly wholegrain and/or high cereal fibre varieties. ^5^ Includes fruit or vegetable juice. ^6^ Vegetables and legumes/beans. ^7^ Lean meats and poultry, fish, eggs, tofu, nuts and seeds and legumes/beans. ^8^ Milk, yoghurt, cheese and/or alternatives, mostly reduced fat.

**Table 4:** Total daily energy and nutrient intake at breakfast, the rest of the day, and the total day by breakfast consumer category.

Nutrient	Total Day			Breakfast		The Rest of the Day		
	Breakfast Cereal Consumers ^1^ (*n* = 3798)	Non-Cereal Breakfast Consumers ^2^ (*n* = 4392)	Breakfast Skippers ^3^ (*n* = 1151)	Breakfast Cereal Consumers ^1^ (*n* = 3798)	Non-Cereal Breakfast Consumers ^2^ (*n* = 4392)	Breakfast Cereal Consumers ^1^ (*n* = 3798)	Non-Cereal Breakfast Consumers ^2^ (*n* = 4392)	Breakfast Skippers ^3^ (*n* = 1151)
	Nutrient Intake * (mean ± SE)							
Energy (MJ)	8.7 ± 0.1 ^a^	8.5 ± 0.1 ^a^	7.9 ± 0.1 ^b^	1.8 ± 0.0 ^a^	1.5 ± 0.0 ^b^	7.0 ± 0.1 ^a^	7.1 ± 0.0 ^a^	7.5 ± 0.1 ^b^
Protein (g)	91.5 ± 0.8 ^a^	90.0 ± 0.8 ^a,b^	85.8 ± 1.2 ^b^	14.1 ± 0.2 ^a^	15.6 ± 0.2 ^b^	78.8 ± 0.8 ^a^	76.2 ± 0.8 ^a,b^	74.2 ± 1.1 ^b^
Total fat (g)	71.1 ± 0.6 ^a^	76.5 ± 0.6 ^b^	76.8 ± 0.9 ^b^	8.1 ± 0.2 ^a^	13.7 ± 0.2 ^b^	65.1 ± 0.6	64.8 ± 0.6	63.0 ± 0.8
*Saturated fat (g)*	26.8 ± 0.3 ^a^	28.7 ± 0.3 ^b^	29.6 ± 0.4 ^b^	3.2 ± 0.1 ^a^	5.2 ± 0.1 ^b^	24.4 ± 0.3	24.2 ± 0.3	24.4 ± 0.4
Total sugars (g)	113.4 ± 1.4 ^a^	103.6 ± 1.4 ^b^	108.4 ± 2.0 ^a,b^	28.7 ± 0.5 ^a^	20.8 ± 0.5 ^b^	86.4 ± 1.3 ^a,b^	84.8 ± 1.3 ^a^	93.2 ± 1.9 ^b^
*Added sugars (g)*	46.3 ± 0.8 ^a^	51.7 ± 0.7 ^b^	62.2 ± 1.4 ^c^	8.3 ± 0.4	8.6 ± 0.3	39.9 ± 0.7 ^a^	44.1 ± 0.7 ^b^	52.1 ± 1.3 ^c^
*Free sugars (g)*	54.3 ± 0.8 ^a^	59.0 ± 0.8 ^b^	68.2 ± 1.5 ^c^	11.9 ± 0.4	11.3 ± 0.4	44.2 ± 0.7 ^a^	48.9 ± 0.7 ^b^	57.2 ± 1.4 ^c^
Carbohydrate (g)	244.5 ± 1.6 ^a^	229.3 ± 1.6 ^b^	226.5 ± 2.4 ^b^	61.7 ± 0.5 ^a^	48.8 ± 0.5 ^b^	186.7 ± 1.5	185.3 ± 1.5	191.7 ± 2.2
Fibre (g)	26.9 ± 0.3 ^a^	22.2 ± 0.3 ^b^	19.4 ± 0.4 ^c^	7.4 ± 0.1 ^a^	4.9 ± 0.1 ^b^	19.5 ± 0.2 ^a^	17.6 ± 0.2 ^b^	16.5 ± 0.3 ^b^
Niacin (equivalents) (mg)	42.2 ± 0.4 ^a^	40.4 ± 0.4 ^b^	39.2 ± 0.6 ^b^	9.1 ± 0.2 ^a^	8.4 ± 0.2 ^b^	33.8 ± 0.4	32.8 ± 0.4	34.0 ± 0.6
Iron (mg)	13.6 ± 0.1 ^a^	10.3 ± 0.1 ^b^	9.1 ± 0.2 ^c^	5.0 ± 0.1 ^a^	2.3 ± 0.1 ^b^	8.7 ± 0.1 ^a^	8.2 ± 0.1 ^b^	7.8 ± 0.1 ^b^
Thiamin (mg)	1.9 ± 0.0 ^a^	1.5 ± 0.0 ^b^	1.1 ± 0.0 ^c^	0.8 ± 0.0 ^a^	0.5 ± 0.0 ^b^	1.1 ± 0.0	1.0 ± 0.0	1.0 ± 0.0
Riboflavin (equivalents) (mg)	2.3 ± 0.0 ^a^	1.7 ± 0.0 ^b^	1.5 ± 0.0 ^c^	1.0 ± 0.0 ^a^	0.5 ± 0.0 ^b^	1.4 ± 0.0 ^a^	1.2 ± 0.0 ^b^	1.3 ± 0.0 ^a,b^
Folate (µg)	707 ± 8 ^a^	595 ± 8 ^b^	465 ± 12 ^c^	253 ± 6 ^a^	208 ± 6 ^b^	449 ± 6 ^a^	396 ± 6 ^b^	424 ± 9 ^a,b^
Calcium (mg)	927 ± 10 ^a^	741 ± 10 ^b^	673 ± 15 ^c^	291 ± 5 ^a^	177 ± 5 ^b^	644 ± 9 ^a^	580 ± 9 ^b^	596 ± 13 ^b^
Sodium (mg)	2283 ± 30 ^a^	2587 ± 29 ^b^	2431 ± 44 ^a^	244 ± 11 ^a^	570 ± 11 ^b^	2075 ± 28	2069 ± 28	2107 ± 41
Magnesium (mg)	371 ± 3 ^a^	327 ± 3 ^b^	301 ± 4 ^c^	98 ± 1 ^a^	69 ± 1 ^b^	276 ± 5 ^a^	264 ± 2 ^b^	259 ± 4 ^b^
Potassium (mg)	3146 ± 25 ^a^	2872 ± 24 ^b^	2665 ± 36 ^c^	646 ± 8 ^a^	520 ± 8 ^b^	2539 ± 23 ^a^	2405 ± 22 ^b^	2319 ± 33 ^b^

Abbreviations: SE—standard error. * Energy intake adjusted for sex, age group, their interaction, and number of eating occasions at breakfast; all other nutrients adjusted for sex, age group, their interaction, BMI group, and energy intake at total day, breakfast, or the rest of the day. ^1^ Breakfast cereal consumers had any food from the sub-major food groups “breakfast cereals, ready to eat” or “breakfast cereals, hot porridge style”, either at any time of day with a “breakfast” eating occasion, or between 5.30 a.m. and 9.30 a.m. with an “extended consumption” eating occasion. ^2^ Non-cereal consumers reported “breakfast” eating occasions but were not breakfast cereal consumers. ^3^ Breakfast skippers did not report a “breakfast” eating occasion. ^a,b,c^ Different superscripts denotes significant difference between groups (*p* < 0.001) by post hoc, Bonferroni.

## Supplementary Materials

The following are available online at http://www.mdpi.com/2072-6643/11/1/175/s1, Table S1: Top 10 sub-major food groups consumed at breakfast ranked by prevalence of consumption among breakfast consumers, Table S2: Top sub-major food groups at breakfast by the contribution to total daily discretionary energy among breakfast consumers., Table S3: Contribution from the breakfast cereal to total daily nutrient intakes.
