# Supplementary material for: Breakfast Choice Is Associated with Nutrient, Food Group and Discretionary Intakes in Australian Adults at Both Breakfast and the Rest of the Day
Source: Nutrients. 2019 Jan 15;11(1):175. doi: 10.3390/nu11010175 (PMC6356876; doi:10.3390/nu11010175)
Supplement: Supplementary file 1 [file nutrients-11-00175-s001.pdf]

Supplementary Table 1 Top 10 sub-major food groups consumed at breakfast ranked by prevalence of consumption among breakfast consumers.

Rank	Breakfast cereal consumers			Non-cereal breakfast consumers		
	Sub-major food group	% consumers	Median grams per consumer	Sub-major food group	% consumers	Median grams per consumer
1	Breakfast cereals, ready to eat	81.7%	47	Regular breads, and bread rolls (plain/unfilled/untopped varieties)	51.6%	56
2	Dairy milk (cow, sheep and goat)	79.2%	142	Dairy milk (cow, sheep and goat)	36.4%	31
3	Sugar, honey and syrups	35.8%	8	Coffee and coffee substitutes	30.9%	220
4	Tea	25.6%	200	Sugar, honey and syrups	28.8%	7
5	Coffee and coffee substitutes	22.5%	200	Tea	22.7%	220
6	Breakfast cereals, hot porridge style	18.9%	203	Margarine and table spreads	21.1%	5
7	Regular breads, and bread rolls (plain/unfilled/untopped varieties)	16.1%	36	Butters	12.4%	7
8	Yoghurt	11.6%	62	Eggs	12.1%	78
9	Fruit and vegetable juices, and drinks	11.4%	263	Yeast, and yeast vegetable or meat extracts	11.2%	6
10	Tropical and subtropical fruit	9.7%	98	Fruit and vegetable juices, and drinks	10.0%	302

Supplementary Table 2 Top sub-major food groups at breakfast by the contribution to total daily discretionary energy among breakfast consumers.

Rank	Breakfast cereal consumers				Non-cereal breakfast consumers			
	Sub-major food group	% contribution	% consumers	kJ among consumers	Food group	% contribution	% consumers	kJ among consumers
1	Sugar, honey and syrups	4.7%	35.8%	204	Sugar, honey and syrups	3.0%	28.8%	161
2	Breakfast cereals, ready to eat	0.8%	2.6%	825	Butters	2.0%	12.4%	323
3	Jam and lemon spreads, chocolate spreads, sauces	0.6%	5.8%	203	Processed meat	1.6%	8.2%	545
4	Fruit and vegetable juices, and drinks	0.6%	2.5%	489	Jam and lemon spreads, chocolate spreads, sauces	1.6%	9.4%	243
5	Butters	0.6%	5.1%	247	Pastries	0.8%	2.2%	1436
6	Other beverage flavourings and prepared beverages	0.3%	1.4%	144	Cakes, muffins, scones, cake-type desserts	0.7%	1.8%	2035
7	Formula dietary foods	0.2%	0.6%	597	Sweet biscuits	0.7%	1.8%	635
8	Processed meat	0.2%	0.9%	610	Yeast, and yeast vegetable or meat extracts	0.6%	11.2%	47
9	Cakes, muffins, scones, cake-type desserts	0.1%	0.3%	1618	Fruit and	0.6%	3.1%	547

					vegetable juices, and drinks			
10	Dairy blends	0.1%	1.0%	219	Muesli or cereal style bars	0.4%	1.3%	733

Supplementary Table 3 Contribution from the breakfast cereal to total daily nutrient intakes.

Nutrient	Contribution to daily intakes (%)
Energy	10.6%
Protein	8.0%
Total fat	5.6%
Saturated fat	3.5%
Total sugars	7.8%
Added sugars	12.5%
Free sugars	11.7%
Carbohydrate	15.3%
Fibre	21.9%
Niacin	13.8%
Iron	32.6%
Thiamin	35.6%
Riboflavin	23.2%
Folate	26.0%
Calcium	9.7%
Sodium	5.4%
Magnesium	16.5%
Potassium	7.9%
